# Impact of social media education on antimicrobial stewardship awareness among pharmacy, medical and nursing students and residents

**DOI:** 10.1186/s12909-023-04423-w

**Published:** 2023-06-16

**Authors:** Stephanie Atallah, Hanine Mansour, Hani Dimassi, Wissam K Kabbara

**Affiliations:** 1 Department of Pharmacy Practice, Lebanese American University (LAU), Byblos, Lebanon; 2 Department of Pharmaceutical Sciences, Lebanese American University (LAU), Byblos, Lebanon

**Keywords:** Antimicrobial stewardship, Education, Pharmacy, Medicine, Nursing, Social media

## Abstract

**Background:**

Antimicrobial resistance has always been an important issue as antimicrobial therapies are becoming less effective due to incorrect use and overuse. Our objective was to evaluate the impact of social media education on spreading antimicrobial stewardship awareness among healthcare students and residents.

**Methods:**

A prospective interventional study was conducted over 5 months, from November 2021 until March 2022. Weekly educational posts on infectious diseases were posted along with pre- and post- quizzes on a designated Facebook page. The primary endpoint of change in knowledge score was assessed using the independent t-test. Expected average pre-training is 2.5 over 5, and the expected average post-training is a minimum of 3.5 over 5 (common standard deviation of 1) for a minimum of 20% improvement that produces an effect size d = 1. Expecting a larger number of respondents on the pre-test than post-test, the ratio N1/N2 was set to 1.5. With the desired power set to 80% and alpha at 5%, sample size was determined as a minimum of 22 (N1) and 14 (N2). All analyses were carried at the 0.05 significance level.

**Results:**

In the entry questionnaire, 85.6% (107/125) of participants believed that antibiotics are overused, 26.4% (33/125) confirmed that they overuse antibiotics, and 88.8% (111/125) confirmed the importance of having an antimicrobial stewardship program. 76.8% (96/125) of the participants regularly use social media for educational purposes and only 2.4% sometimes refer to social media as an educational tool. Improvement in knowledge was noted in all pre and post – quizzes except for two quizzes (prostatitis and acute cystitis – 18.4% and 13.2% improvement respectively). In total, there was a significant 36.2% improvement between all pre and post quizzes [min 13.2% and max 52.8% across all quizzes].

**Conclusion:**

This intervention demonstrated the importance of social media as a valuable tool to enhance antimicrobial stewardship knowledge among pharmacy, medical and nursing students and residents. Future studies are needed to examine the impact of social media education on behaviors in practice.

## Background

Despite several published guidelines, inappropriate prescribing has been reported as the main cause for antimicrobial resistance [1]. The exponential increase in antimicrobial resistance worldwide limited the use of many existing antibiotics and increased the development of co-infections such as antimicrobial associated *Clostridioides difficile* infection (CDI) [1, 2]. With very few new antibiotics in the pipeline, patients’ hospitalization and death due to infections like methicillin resistant *Staphylococcus aureus* (MRSA) are increasing [2]. Antibiotic malpractice led to the development of antimicrobial stewardship programs (ASP) that focus on optimizing antimicrobial use to improve prescribing patterns [1]. One of the important core elements of the ASP is education of prescribers through orientations, continuing education and presentations [3]. ASP also focuses on creating local antibiotic policy guidance while referring to World health organization (WHO) manual. This strategy of education has shown to improve patient care [4]. Studies have shown that patients have greater in - hospital mortality rates if they have received inappropriate initial antimicrobial coverage [5]. In 2013, the Centers for Disease Control and Prevention (CDC) stated that at least 2 million infections and 23,000 deaths are due to antimicrobial resistant infections in the United States every year [6]. Mitchell et al. demonstrated that antibiotic - resistant organisms are common news among mainstream media outlets [6]. Given the complexity of antimicrobial agents, changing guidelines recommendations, and varying institution - specific bacterial epidemiology, optimization of antimicrobial therapy constitutes a clinical challenge [4].

In Beirut, Lebanon, most healthcare students belong to the “Net generation”, where their ages range between 18 and 25 years old [7]. Studies show that online education through social media is growing due to the time restrictions that most of these students are facing [4]. Social media evolution is creating a new venue for rapid dissemination of information, advocacy on critical issues, and education to a broader audience [8]. Education of young healthcare professionals today demands innovative strategies beyond the lecture hall [3]. Social media platforms, such as Facebook and twitter may be an effective tool when used to educate prescribers about the Infectious Diseases Society of America (IDSA), Centers for Disease Control and Prevention (CDC) and other local treatment guidelines [6]. Few other studies have demonstrated that technology increases the number of antimicrobial interventions by significantly reducing the time in both de-escalation and escalation to appropriate antimicrobial therapy [9, 10]. Pisano et al. was the first study to add evidence to the literature concerning the beneficial influence of social media intervention on knowledge of antimicrobial prescribing patterns [4]. They emphasized on the consequences of unnecessary use of antibiotics, including the increase in antimicrobial resistance and the harm of antibiotics related toxicities. Also, this study was able to prove that social media might be a valuable tool to reach medical students and residents. This tool might be able to disseminate and reinforce important information regarding antibiotics treatment choices and consequently improve prescribing patterns [4].

However, there is no conclusive data that proves social media education is the major factor contributing to the improvement in students’ knowledge and confidence in prescribing. Further studies need to be conducted to assess the true influence of social media education on young health care prescribers [4]. The objective of this study was to evaluate the impact of using Facebook, as a social media platform, on spreading antimicrobial stewardship awareness among healthcare students and residents.

## Methods

### Setting and subjects

This study was conducted over five months, from November 2021 until March 2022. It is a prospective interventional study done by opening a Facebook page known as “Bug Busters”. The page was public and categorized as an educational website on Facebook. Participants contacted were defined as medical (medical year 3 or year 4 students or residents), pharmacy (pharmacy year 3 or year 4 students or residents) and senior nursing students. At the beginning of the study, requests to participate in the study were sent to all the schools of medicine, pharmacy and nursing in Beirut, Lebanon. Mainly the deans of the schools or the deans’ office administrators’ assistants were contacted via email at first, and later a link to the google form containing the consent form was shared with them also via email. Participants completing their experiential education in Beirut, Lebanon were included. Participants who did not sign the consent form were excluded. The consent form was obtained and documented as a written statement describing the research and was archived in the research records.

### Data collection

An entry questionnaire was shared as a link to a google form via email to all participants; it was also posted on our Facebook page. The entry questionnaire collected some background and demographic data about the participants. It included questions that evaluated their frequency of social media use, antimicrobial practice knowledge and their belief about the importance of antimicrobial overuse, resistance and stewardship programs. In addition, the questionnaire helped us gather information about participants’ age, gender, occupation, university attended and school year. At the end of the intervention period, an exit questionnaire was shared as a link to a google form to all participants and was also posted on our Facebook page. This exit questionnaire helped us assess the increase in prescribing confidence of the participants and identify if any references other than the shared educational posts were used.

A five - question pre - quiz was posted on the Facebook page every Monday, one day before the educational post. A five - question post - quiz was posted on the Facebook page every Friday, three days after the educational post. The post – quiz consisted of the same questions as the pre – quiz to assess the change in knowledge score. All quizzes were multiple choice and each question was independent from the other. Students participating in the weekly quizzes served as their own control since pre - quizzes and post - quizzes were done. The educational posts were posted on the Facebook page every Tuesday; they followed the WHO – ASP (World Health Organization – Antimicrobial Stewardship Program) competencies toolkit in health – care facilities.^2^ The posts included questions regarding knowledge in prescribing principles such as appropriate antibiotic indication, empiric treatment choice and proper de-escalation. In addition, questions regarding correct dosing regimens, renal dose adjustments, pharmacokinetics, allergies, intravenous to oral conversion and different antibiotic drug classes’ side effects were included. The quizzes were posted on the Facebook page as links to google forms. Google forms revealed the quiz questions with wrong answers but did not indicate its’ correction. Each quiz contained a variety of questions ranging from basic knowledge to advanced clinical thinking. The educational posts were posted as pictures on the Facebook page. Quizzes and posts were reviewed by two infectious diseases clinical faculty pharmacists prior to posting.

### Statistical analysis

Expected average pre-training is 2.5 over 5, and the expected average post-training is a minimum of 3.5 over 5 (common standard deviation of 1) for a minimum of 20% improvement (at least 1 score improvements out of a total of 5) that produced an effect size d = 1. Expecting a larger number of respondents on the pre-test than post-test, the ratio N1/N2 was set to 1.5. Desired power was set to 80%, and alpha at 5%, sample size was determined as 22 (N1) and 14 (N2). A target of more than 300 students or residents were contacted to account for possible refusal to participate, missing information and data loss. Data was coded and exported into SPSS V26 for analysis.

### Outcomes

The primary endpoint of change in knowledge score was assessed using the independent t - test. The secondary endpoints included factors affecting the change in knowledge score; pre and post test were compared for each test as well as for each question type using the Pearson chi-square test. All analyses were carried at a 0.05 significance level.

## Results

From November 2021 until March 2022, a total of 125 participants consented to participate in the study. The baseline characteristics of the participants demonstrated that 71.2% were females, 63.2% were pharmacy major, 25.6% medical major and 11.2% nursing major. The total duration of the assigned follow-up was 151 days. The mean population age was 23.4 years [min 21 years – max 29 years]. Participants attended different universities in Beirut, Lebanon including Lebanese American University (LAU) 75.2% (94/125), American University of Beirut (AUB) 7.2% (9/125), Holy Spirit University Kaslik (USEK) 4.8% (6/125), Lebanese International University (LIU) 2.4% (3/125), Notre Dame University (NDU) 2.4% (3/125), Beirut Arab University (BAU) 2.4% (3/125), University Saint Joseph (USJ) 1.6% (2/125), Balamand University 0.8% (1/125), Makassed University of Beirut 0.8% (1/125), Lebanese University (LU) 0.8% (1/125). Two participants were from foreign universities: Bahauddin Zakariya University Multan Pakistan 0.8% (1/125) and Tishreen University 0.8% (1/125). Participants were divided as follows: 3.2% (4/125) professional year-2 pharmacy students, 24.8% (31/125) professional year-3 pharmacy students, 30.4% (38/125) professional year-4 pharmacy students, 1.6% (2/125) masters, 0.8% (1/125) community pharmacist, 12% (15/125) medical year-3 students, 8.8% (11/125) medical year-4 students, 11.2% (14/125) nursing students and 7.2% (9/125) residents (of whom 33.33% (3/9) were pharmacy residents and 66.66% (6/9) were medical residents). 40.8% (51/125) participants have an infectious diseases or antimicrobial stewardship rotation before the end of the study in March. In the entry questionnaire, 85.6% (107/125) believed that antibiotics are overused, 26.4% (33/125) confirmed that they overuse antibiotics, and 88.8% (111/125) confirmed the importance of having an antimicrobial stewardship program. Table 1 represents the participants’ characteristics.

**Table 1:** Participants’ characteristics

Criteria	Number	Percentage %
Female	89/125	71.2
Pharmacy major	79/125	63.2
Medical major	32/125	25.6
Nursing major	14/125	11.2
ID or AMS rotation	50/125	40.8
Believed antibiotics are overused	107/125	85.6
Confirmed they overuse antibiotics	33/125	26.4
Confirmed the importance of an AMS program	111/125	88.8
Use social media for educational purposes	96/125	76.8
Sometimes refer to social media as an educational tool	3/125	2.4

76.8% (96/125) regularly use social media for educational purposes and 2.4% sometimes refer to social media as an educational tool. The most common social media platform used was Instagram 63.2% (79/125) followed by Facebook 56% (70/125), LinkedIn 43.2% (54/125), YouTube 40.8% (51/125), Twitter 29.6% (37/125) and finally Medscape 2.4% (3/125). Table 2 represents the different social media platforms used by the participants for educational purposes.

**Table 2:** Representation of the different social media platforms used by the participants for educational purposes

Participants that use social media (N = 99/125)		
Platform preferred	Number	Percentage%
Instagram	79/125	63.2
Facebook	70/125	56.0
LinkedIn	54/125	43.2
YouTube	51/125	40.8
Twitter	37/125	29.6
Medscape	3/125	2.4

Improvement in knowledge (> 20% improvement) was noted in all pre and post – quizzes except for two quizzes prostatitis and acute cystitis – 18.4% and 13.2% improvement respectively. In total, there was a 36.2% improvement between all pre and post quizzes [min 13.2% and max 52.8% across all quizzes]. However, not all quizzes showed significant improvement results. Non-significant improvement results were respectively for cellulitis, community acquired pneumonia, prostatitis, acute cystitis and acute cystitis treatment; 30.8% (p = 0.073), 21.6% (p = 0.231), 18.4% (p = 0.057), 13% (p = 0.125) and 20.8% (p = 0.098). Table 3 represents the improvement in knowledge scores between pre and post quizzes.

**Table 3:** Improvement in knowledge scores between pre and post quizzes^*^

Quiz	Type of infection	Average Pre-Quizzes			Average Post-Quizzes			% Improvement		
		N^1^	Mean	SD	N^1^	Mean	SD		P-value	
**1**	Cellulitis	13	2.46	1.45	4	4.00	1.15	30.8	0.073	
**2**	CAP^2^	4	3.25	0.95	3	4.33	1.15	21.6	0.231	
**3**	CAP drug SEs^3^	7	2.43	0.97	10	3.60	1.07	23.4	0.037	
**4**	Prostatitis	36	2.08	1.10	7	3.00	1.29	18.4	0.057	
**5**	Acute cystitis	18	2.89	1.13	13	3.54	1.12	13.0	0.125	
**6**	Acute cystitis treatment	14	2.79	1.31	6	3.83	0.98	20.8	0.098	
**7**	Meningitis treatment	9	2.56	0.72	3	4.67	0.57	42.2	0.001	
**8**	*Clostridiodes difficile*	51	2.35	1.05	28	4.04	0.99	33.8	< 0.001	
**9**	Infective endocarditis	27	2.48	0.80	24	4.67	0.48	43.8	< 0.001	
**10**	Native Valve Endocarditis	29	1.93	0.88	23	4.57	0.50	52.8	< 0.001	
**11**	Prosthetic Valve Endocarditis	17	2.82	0.72	15	4.67	0.48	37.0	< 0.001	
**12**	HAP^4^	23	2.74	0.91	25	4.48	0.82	34.8	< 0.001	
**13**	HAP / VAP^5^ treatment	20	2.40	0.94	30	4.23	1.04	36.6	< 0.001	
**14**	HAP treatment	26	2.50	0.86	25	4.32	1.10	36.4	< 0.001	
**15**	Intra-abdominal infection	23	1.96	1.18	22	4.27	0.93	46.2	< 0.001	
**16**	Intra-abdominal infection II	21	2.62	0.74	17	4.35	0.78	34.6	< 0.001	
**17**	Contiguous osteomyelitis	17	2.82	0.80	18	4.61	0.50	35.8	< 0.001	
**18**	Hematogenous Osteomyelitis	24	2.75	1.22	21	4.14	0.96	27.8	< 0.001	
**Total**		24	2.46	1.03	21	4.27	0.93	36.2	< 0.001	

1 Number of participants who completed the quiz

2 Community-acquired pneumonia

3 Side effects

4 Hospital-acquired pneumonia

5 Ventilator-associated pneumonia

* Data was analyzed using the independent samples t-test

Improvement in knowledge was significantly seen across all questions types (symptoms, duration of therapy, disease specific micro-organism, disease specific drug of choice, drug dose, adverse effects, pharmacokinetics, IV to PO conversion and prescribing knowledge). Table 4 represents the improvement in knowledge scores between pre and post quizzes based on the type of question.

**Table 4:** Improvement in knowledge scores per question type between pre and post quizzes

	% Correct Pre-Quizzes		% Correct Post-Quizzes		% Improvement	P-value
	n/N*	Mean%	n/N	Mean%		
**Diagnosis**	74/135	54.8%	68/86	79.1%	24.3%	< 0.001
**Symptoms**	30/82	36.6%	48/62	77.4%	40.8%	< 0.001
**Duration of therapy**	50/143	35.0%	106/126	84.1%	49.1%	< 0.001
**Disease specific micro-organism**	69/171	40.4%	98/111	88.3%	47.9%	< 0.001
**Disease specific drug of choice**	220/527	41.7%	296/358	82.7%	41.0%	< 0.001
**Drug dose**	164/280	58.6%	252/280	90.0%	31.4%	< 0.001
**Adverse effects**	17/35	48.6%	36/50	72.0%	23.4%	0.028
**PK/IV to PO**	125/219	57.1%	126/147	85.7%	28.6%	< 0.001
**Prescribing knowledge**	183/302	60.6%	226/250	90.4%	29.8%	< 0.001

* n is the number of correct answers/N is the total number of questions per question type

Regarding the factors influencing the results, 40.8% (51/125) of the participants confirmed having an infectious disease or antimicrobial stewardship rotation during the intervention period. In addition, only 4% (3/75) mentioned using platforms other than the educational posts to answer the quizzes

## Discussion

Antimicrobial resistance (AMR) is one of the most serious global public health issues today. Antibitoic-resistant infections, longer hospital stays, higher medication costs, an overburdened public health system, service delays, and higher mortality rates have all been linked to AMR. The discovery of new antibiotics has slowed dramatically in recent decades. Furthermore, AMR reduces the efficacy of current antibiotics. The World Health Organization (WHO) recently released a study titled “Global Antimicrobial Resistance and Use Surveillance System (GLASS) Report: Early Deployment 2020,“ which revealed troubling AMR rates in 78 countries. The focus now is on the correct antibiotic, for the correct indication, the correct condition, at the correct time, with the correct dosage, path, and length of therapy, with the least amount of risk to the patient and potential patients. Through incorporating evidence-based approaches, an ASP has consistently shown an increase in the safe and effective use of antimicrobials [11].

Most students and residents acknowledged that antibiotic resistance was a challenge and recognized the value of good-antibiotic-use knowledge. However, only 26.4% of the participants confirmed that they overuse antibiotics. The exit questionnaire confirmed the importance of antimicrobial stewardship programs and highlighted the increase in confidence and usefulness of the educational posts. A small number of participants still referred to other references such as Medscape; however, this referral didn’t influence the impact of the educational posts on the gained knowledge.

Instagram and Facebook were the most commonly used social media platforms by the participants in this study. The use of Instagram might have exceeded that of Facebook due to the more user-friendly features offered by Instagram. This might be because Instagram has the poll feature which makes learning easier compared to accessing the quizzes via the link on the Facebook page.

Pisano et al. (2019) conducted an interventional study where internal medicine residents at their academic center were offered iPads to access available ASP tools and enter trivia contests on Facebook and Twitter for better dissemination of information. In addition, the authors tested if social media reinforcement would also increase the use of the hospital’s order sets. Confidence in choice did not significantly change pre and post intervention; however, confidence in choosing the appropriate duration of antibiotics increased significantly. Residents also gained wide knowledge about ASP and drug dosing recommendations. All knowledge-based test scores significantly improved and those who had an ID rotation did not have better scores than the others. These results are consistent with our study; nevertheless, it is important to note that our study was anonymous, had a wider range of participants (not only medical residents) and did not include the use of hospital order sets. Our main objective was to use social media to disseminate antimicrobial stewardship knowledge.

Our research study had some limitations. First, we had a relatively small sample size in few of the quizzes which subsequently were not powered to detect a difference. However, effect size was achieved as the under-powered quizzes showed at least 20% improvement in quiz scores even if the p-value was not statistically significant (cellulitis, community acquired pneumonia and acute cystitis treatment). Therefore, clinical significance in these topics was achieved even if statistical significance was not reached. Second, the participants in the pre-intervention phase may not have been exactly the same as that in the post-intervention phase since the quizzes shared on the Facebook page were filled anonymously. The participants demographic information was independently collected from the quizzes and cannot be tracked by using the Facebook social media platform due to privacy/anonymity of answers Third, some students / residents had completed an infectious disease or antimicrobial stewardship rotation during the post-intervention phase. This might have interfered with the study results where participants might have scored higher in the post intervention quizzes. Fourth, we had two participants that were not from Lebanese universities and we could not trace their results due to anonymous reasons so excluding them was not possible. In addition, many students/residents are not using the Facebook platform anymore and reaching them can be challenging.

This interventional study allowed the investigators to interact with many healthcare professionals and students/residents including community pharmacists, healthcare students from different majors, masters’ students and residents from outside Lebanon. Many professionals followed the Facebook page and interacted on the educational posts without filling out the quizzes due to inclusion criteria restrictions. The educational posts are always available for referral and can be saved as pictures to ensure maximal benefit for the interested audience.

Social media education might be more effective than expected especially during these times where technology is being integrated more in the medical field [1]. This is strongly supported now with the COVID-19 pandemic where all education has shifted to online learning and social media’s role is expanding. The extensive usage and deployment of social media in medical education is not currently included in most health schools’ curricula [12]. By offering a common channel for healthcare professionals, patients, and the general public to communicate about health concerns, social media adds a new dimension to healthcare with the potential to enhance health outcomes. It’s a useful tool for social engagement and continual education, and it encourages user participation. In times of the pandemic, where temporal urgency, geographic distance, and the need to extensively spread knowledge have driven us to develop new ways of working and learning, there are clear benefits to using social media for health communication. However, when participating in healthcare discussions it is critical that social media users monitor the material transmitted for quality and dependability, as well as the maintenance of patient confidentiality [13].

## Conclusion

Social media plays a major role as an educational tool for the new healthcare generation. This interventional study demonstrated the involvement of healthcare students/residents in online education and the importance of social media platforms as a venue for antimicrobial stewardship learning. Further studies need to be conducted in different disciplines to evaluate the effectiveness of social media education and its’ impact on healthcare practice.

## Data Availability

The datasets used and/or analyzed during the current study are available from the corresponding author on reasonable request.
